# Targeting the latest site of left ventricular mechanical activation is associated with improved long-term outcomes for recipients of cardiac resynchronization therapy

**DOI:** 10.1016/j.hroo.2022.05.003

**Published:** 2022-05-13

**Authors:** Rasmus Borgquist, William R. Barrington, Zoltan Bakos, Anna Werther-Evaldsson, Samir Saba

**Affiliations:** ∗ Arrhythmia Section, Department of Cardiology, Lund University, Skane University Hospital, Lund, Sweden; † Department of Medicine, University of Pittsburgh Medical Center, Pittsburgh, Pennsylvania; ‡ Department of Cardiology, Kristianstad Hospital, Kristianstad, Sweden; § Heart Failure and Transplant Section, Department of Cardiology, Lund University, Skane University Hospital, Lund, Sweden

**Keywords:** Cardiac resynchronization therapy, Latest mechanical activation, Heart failure hospitalization, Mortality

## Abstract

**Background:**

Previous studies have suggested that targeting the site of latest mechanical activation of the left ventricle (LV) results in improved cardiac resynchronization therapy (CRT) outcomes. It is not known whether these benefits are sustained over medium-term follow-up.

**Objective:**

To assess the clinical outcome of imaging-guided LV lead position.

**Methods:**

We sought to assess the medium-term clinical outcome by performing a patient-level meta-analysis of 2 previously published randomized controlled trials (the “STARTER” trial and the “CRT Clinic” trial). These 2 trials compared imaging-guided LV lead placement in the latest activated scar-free segment (intervention group) to standard of care (control). Mortality and heart failure hospitalization outcomes over extended follow-up were gathered from the medical records and merged. Results were stratified for native electrocardiogram (ECG) morphology.

**Results:**

A total of 289 patients were followed for a median of 6.3 years. Seven years post implant, 47 (28%) in the intervention group had died, vs 47 (38%) in the control group (*P* = .13); 49 (30%) vs 53 (42%) had been hospitalized for heart failure (*P* = .035); and 47% vs 59% (*P* = .057) had reached the combined endpoint. In Kaplan-Meier analysis, patients in the intervention group had better survival free of heart failure hospitalization (*P* = .045) and lower risk of heart failure hospitalization (*P* = .019).

**Conclusion:**

Targeting the latest mechanically activated segment in CRT results in better medium-term clinical outcome, mainly driven by a reduced risk of hospitalization for heart failure. The effect was seen regardless of native ECG morphology.

**Key Findings**

▪ Imaging-guided left ventricular (LV) lead placement targeting the latest mechanically activated segment is feasible and results in a higher proportion of LV leads placed concordant to, or adjacent to, the latest mechanically activated segment.
▪ Early benefits in reverse remodeling for patients with targeted LV lead placement in the latest mechanically activated segment transform into medium-term benefit in hard clinical endpoints.
▪ Targeting the latest mechanically activated segment for LV lead placement in cardiac resynchronization therapy reduces medium-term risk of heart failure hospitalization and mortality.

## Introduction

Cardiac resynchronization therapy (CRT) is an effective treatment for reducing mortality and risk for heart failure hospitalization in patients with systolic heart failure and wide QRS complex.^1^, ^2^, ^3^ The rationale for resynchronization is that by placing the left ventricular (LV) lead on the free wall of the left ventricle, earlier and more synchronized activation of the ventricle is achieved. However, there may be several anatomical options for placing the LV lead in a suitable epicardial vein, and it is unclear what is the best selection method for choosing the final pacing site. Previous observational data have suggested that a lateral, nonapical position may be preferable, but this has not been proven prospectively.^4^, ^5^, ^6^, ^7^, ^8^

In principle, LV lead position can be chosen based on anatomical location, maximized interlead distance from the right ventricular (RV) lead, late electrical activation, or late mechanical activation. In addition, lead stability and local pacing properties are important, since scar tissue, high thresholds, and diaphragmatic stimulation all need to be avoided.^6^^,^^9^^,^^10^ Targeting the mechanically latest activated LV segment is theoretically appealing, since it enables early activation of the part of the ventricle that is contracting latest during intrinsic activation. High-frame-rate imaging is needed to correctly discriminate between the timing of contraction of the various LV segments, and echocardiography is therefore the most suitable modality. Four randomized controlled trials (RCTs) have used speckle-tracking radial strain for evaluation of mechanical activation timing and guiding LV lead placement—the TARGET, STARTER, Imaging CRT, and CRT Clinic studies.^11^, ^12^, ^13^, ^14^

However, there is so far no published data to show if targeting the latest LV site of mechanical activation leads to better medium-term clinical results. We therefore sought to evaluate this by looking at extended data from 2 of the above-mentioned randomized studies, the STARTER and CRT Clinic trials (clinical trials identifier NCT00156390 and NCT01426321, respectively). The research reported in this paper was approved by the local ethics committees and adhered to the Helsinki Declaration.

## Methods

The methodology of both studies has been published previously.^12^^,^^14^ In brief, patients fulfilling guideline indications for CRT treatment (LV ejection fraction [LVEF] ≤35%, QRS >120 ms, NYHA class II–IV, optimal medical therapy) were recruited and randomly assigned to image-guided LV lead placement vs standard of care. Both studies used GE Echopac (Vivid 7 or E9; GE Medical, Horten, Norway) for analysis of radial strain. Speckle tracking–based strain analyses were performed using short-axis views of the mid and basal segments of the left ventricle, leaving the apical segments out. Frame rate was set at between 60 and 90 frames per second, and all images were collected at 3 heart cycles and stored digitally for off-line analysis. The segment with the latest mechanical activation was chosen as the optimal segment. Segments with scar were avoided in both studies. In the STARTER study, scar was defined as thin-walled segments (≤5 mm) with hyperacoustic appearance. In the CRT Clinic study scar was defined either by cardiac magnetic resonance imaging (CMR) showing accumulation of gadolinium contrast, or (in the absence of CMR) by peak radial strain ≤9.5%.

Prior to the implant procedure, the implanting physician was presented with the imaging data results, including which segment should be targeted for patients in the intervention group. For patients in the control group, imaging data were not available to the implanting physician, and the LV lead was targeted at the discretion of the implanter, as standard of care. Patients received a CRT-defibrillator or CRT-pacemaker; no patients had a secondary prevention indication for an implantable cardioverter-defibrillator. A right atrial lead was placed in the right atrial appendage, and an RV lead was placed in the RV apex or in the interventricular septum.

Determination of LV lead concordance was done using a combination of fluoroscopy or chest radiograph in the right anterior oblique view (long axis) and left anterior oblique view (short axis). The segment of the active pacing electrode (cathode) was determined and noted in a bull’s-eye plot, similar to the preoperative plotting of the optimal segment. Pacing in the preoperatively determined optimal segment was considered “concordant LV lead placement.” If pacing was performed in a segment immediately neighboring to the optimal segment (including diagonally), it was considered “adjacent.” Segments with no contact to the optimal segment were considered “remote.” Both studies were approved by the respective local institutional review committee, and the subjects gave written informed consent.

Initial follow-up included an echocardiogram and clinical evaluation after 6–12 months. Positive reverse remodeling response was defined as an LVEF improvement of 5% or more. Positive clinical response was considered if there was an improvement of at least 1 NYHA class, in the absence of heart failure hospitalization. For the present study, a review of medical records and mortality registry data was performed by a physician blinded to the study group allocation. Date for first hospitalization for heart failure (as main diagnosis) or death was recorded. Data regarding hospitalization, death, and mode of death were retrieved from the medical records by 1 (CRT Clinic cohort) or 2 (STARTER cohort) blinded clinicians and cross-checked with the population registry (CRT Clinic cohort). These new outcome data were then merged with the original dataset. Primary endpoint was a composite of freedom from heart failure hospitalization and death, and secondary endpoints were each of the components assessed individually. The research reported in this paper adhered to the Helsinki Declaration guidelines.

### Statistical methods

Data analysis was performed with SPSS (Version 27; IBM, Armonk, NY). Continuous variables are expressed as mean ± standard deviation or median [interquartile range, IQR]. Categorical variables are presented as percentages. Differences between groups were assessed using Student *t* tests for continuous variables and Mann-Whitney *U* test, 1-way ANOVA, χ^2^ test, or Fisher exact test for ordinal variables, as appropriate. We used the Kaplan-Meier method to estimate the cumulative hospitalization and death rates, both for the primary groups and as a secondary on-treatment analysis. The assumption of proportional hazard was tested using visual inspection of the curves. Stratified log-rank statistic was used to compare groups. Cox regression was used to evaluate continuous or multinomial predictors of clinical outcome. A *P* value of <.05 was considered statistically significant.

## Results

A total of 289 patients at the 2 sites were randomized to image-guided intervention (n = 163) or standard of care (n = 126) and were followed for a median of 6.3 [IQR 3.6–8.5] years. Baseline characteristics are presented in Table 1 and did not differ between groups. The majority of patients were male (n = 214, 74%), had symptoms corresponding to NYHA class III (n = 191, 66%) and had left bundle branch block morphology on electrocardiogram (ECG) (n = 205, 71%).

**Table 1:** Demographic data and lead positions

	Control N = 126	Image-guided LV lead placement group N = 163	*P* value
Age, years	67 ± 11	66 ± 10	.24
Female sex, n (%)	29 (23)	47 (29)	.35
Ischemic cardiomyopathy, n (%)	76 (60)	77 (47)	.06
ECG morphology, n (%)			.75
LBBB	88 (70)	117 (72)	
Paced	24 (19)	28 (17)	
Non-LBBB	14 (11)	18 (11)	
QRS duration (ms)	165 ± 25	162 ± 25	.31
Diabetes, n (%)	37 (29)	47 (29)	1.0
Renal failure, n (%)	13 (10)	11 (7)	.40
Atrial fibrillation, n (%)	33 (26)	51 (31)	.43
Beta-blocker therapy, n (%)	105 (83)	148 (91)	.07
ACEi or ARB therapy, n (%)	113 (90)	153 (88)	.70
Aldosterone antagonist therapy, n (%)	39 (31)	51 (31)	.97
Loop diuretic therapy, n (%)	98 (78)	116 (71)	.43
NYHA class, n (%)			.37
II	19 (15)	33 (20)	
III	82 (65)	106 (65)	
IV	25 (20)	24 (15)	
LVEF, % [IQR]	25 [20–30]	25 [20–29]	.59
LVESV, mL [IQR]	138 [112–183]	147 [106–185]	.77
LVEDV, mL [IQR]	189 [155–234]	196 [145–234]	.96
Follow-up time, years [IQR]	6 [3.4–8.9]	6.5 |3.7–8.7]	.40
Biventricular pacing, % [IQR]	99 [97–99]	99 [98–99]	.88
Left ventricular lead placement, n (%)			.004
Concordant	18 (14)	44 (27)	
Adjacent	70 (56)	93 (57)	
Remote	38 (30)	26 (16)	

ACEi = angiotensin-converting enzyme inhibitor; ARB = angiotensin receptor antagonist; LV = left ventricular; LBBB = left bundle branch block; LVEDV = left ventricular end-diastolic volume; LVEF = left ventricular ejection fraction; LVESV = left ventricular end-systolic volume; NYHA = New York Heart Association classification of heart failure.

**Figure 1:**
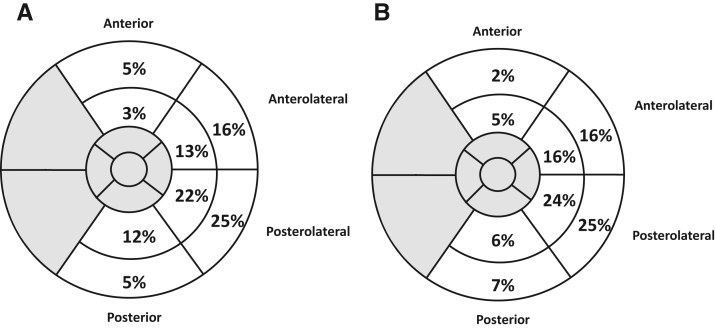
Optimal left ventricular lead locations as indicated by radial strain, shown in a bull’s-eye plot for the 2 groups (control and intervention). Basal segments are shown in the outer circle, mid segments in the middle circle, and apical segments in the innermost 2 circles. Segments in gray are septal or apical in location and were not used for strain analysis. Numbers represent percentage of cases where the optimal segment was found in that location. **A:** Control group. **B:** Intervention group.

Echocardiography strain evaluation indicated that the optimal segment was most often located in the posterolateral part of the left ventricle, but in a substantial proportion of patients another segment was indicated as the latest activated (Figure 1). Patients in the image-guided group were more likely to have the LV lead placed in the optimal or an immediately adjacent segment (134 [84%] in the intervention group vs 88 [70%] in the control group, *P* = .004, Table 1).

Clinical outcome parameters are presented in Table 2. LVEF improved by a median of 8% [IQR 0%–16%] in the total cohort, 9% [IQR 1%–18%] in the intervention group vs 7% [IQR 0%–14%] in the control group, *P* = .24. Image-guided intervention resulted in a numerically higher proportion of echocardiographic responders at 6–12 months (defined as ≥5% absolute increase in LVEF compared to baseline), 103 (63%) in the intervention group vs 71 (56%) in the control group, but this difference was not statistically significant (*P* = .34). Symptoms improved by 0.9 ± 0.7 NYHA class on average, with 223 (77%) patients showing improvement of at least 1 NYHA class (n = 127 [78%] in the intervention group vs n = 95 [75%] in the control group, *P* = .24).

**Table 2:** Clinical outcome

	Control N = 126	Image-guided LV lead placement group N = 163	*P* value
LVEF improvement ≥5%	71 (56%)	79 (63%)	.34
Hospitalized for heart failure	57 (45%)	61 (37%)	.19
Died	68 (54%)	81 (50%)	.48
Cardiac cause	23 (34%)	14 (17%)	.01
Noncardiac cause	5 (7%)	16 (20%)	
Unknown cause	40 (59%)	51 (63%)	
Hospitalized for heart failure or died	83 (67%)	102 (63%)	.54

LV = left ventricular; LVEF = left ventricular ejection fraction.

During the entire follow-up period 149 (52%) patients died, 118 (41%) were hospitalized for heart failure, and 185 (64%) were either hospitalized for heart failure or died. The cause of death was known in 58 of the 149 deceased patients. Among those, heart failure was the most common cause of death (30/58) and cardiac death (heart failure, arrhythmia) was significantly less common in the intervention group than in the control group (47% vs 82%, *P* = .007).

**Figure 2:**
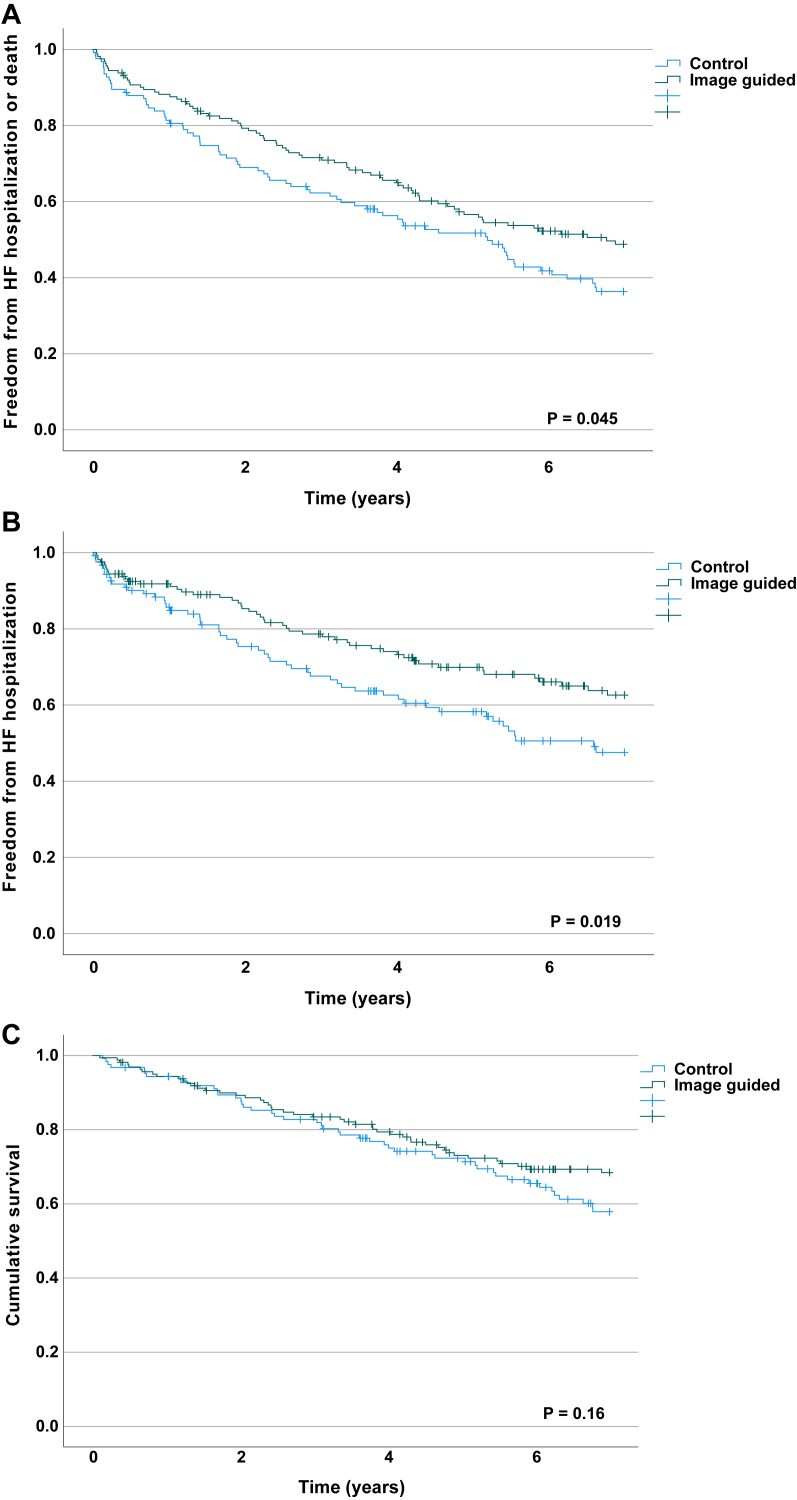
Kaplan-Meier curves showing **A:** survival free of heart failure (HF) hospitalization, **B:** freedom from HF hospitalization, and **C:** overall survival.

Seven years post implant, 47 (28%) patients in the intervention group vs 47 (38%) in the control group (*P* = .13) had died, 49 (30%) vs 53 (42%) (*P* = .035) had been hospitalized for heart failure, and 77 (47%) vs 73 (59%) (*P* = .057) had experienced the combined endpoint. In time-dependent survival analysis there was a significantly higher risk for reaching the combined endpoint of all-cause mortality or heart failure hospitalization (hazard ratio [HR] 1.39 confidence interval [CI 1.01–1.91], *P* = .046) or hospitalization for heart failure (HR 1.59 [CI 1.07–2.34], *P* = .020), but for all-cause mortality alone there was no significant difference (HR 1.33 [CI 0.89–2.00], *P* = .16; see Figure 2 for Kaplan-Meier curves).

In subgroup analysis focusing on different ECG morphologies, the relation between latest activated segment and baseline ECG appearance was further explored. The percentage of left bundle branch block (LBBB) was similar in both groups (70% vs 72%), and ECG morphology was not in itself a predictor of clinical outcome (*P* = .58), whereas QRS duration (HR 0.93 [CI 0.87–0.99] per 10 ms increase in QRS duration) and ischemic etiology (HR 2.3 [1.6-3.2]) were. Numerically, patients with paced QRS were less likely to have the latest activated segment in an inferior location, whereas those with LBBB and non-LBBB native QRS had a similar distribution of segments (Table 3). The success rate in targeting the latest activated segment was similar in the intervention group regardless of the ECG morphology, but in the control group fortuitous optimal/adjacent placement was numerically more common for those with paced QRS pre-CRT. In multivariate Cox regression analysis (adjusting for age, sex, NYHA class, etiology, atrial fibrillation, diabetes, and ECG morphology) imaging-guided strategy was not an independent predictor of outcome (HR 0.88 [CI 0.62–1.2], *P* = .41).

**Table 3:** Left ventricular lead location stratified by electrocardiogram morphology

	LBBB (N = 186)	Paced (N = 48)	Non-LBBB (N = 31)	*P* value
Optimal lead location				.19
Anterior	11 (6%)	5 (10%)	3 (9%)	
Anterolateral	60 (31%)	16 (31%)	10 (31%)	
Posterolateral	93 (47%)	28 (55%)	13 (41%)	
Inferior	33 (17%)	2 (4%)	6 (19%)	
				
Success rate for LV lead positioning				
*All patients*				.42
Concordant or adjacent	145 (78%)	41 (85%)	23 (74%)	
Remote	41 (22%)	7 (16%)	8 (26%)	
*Image-guided group*				.85
Concordant or adjacent	89 (86%)	23 (88%)	14 (82%)	
Remote	15 (14%)	3 (12%)	3 (18%)	
*Control group*				.38
Concordant or adjacent	56 (68%)	18 (82%)	9 (64%)	
Remote	26 (32%)	4 (18%)	5 (36%)	

LV = left ventricular.

## Discussion

In this aggregated pool of 289 patients from 2 RCTs, we show that during a 7-year follow-up, targeting of the latest mechanically activated LV segment resulted in better clinical outcome. While this effect was primarily driven by a reduction in heart failure hospitalizations, the combined endpoint of survival free from heart failure hospitalization was also significantly improved for the intervention group.

### Prior published trials

Two additional studies have evaluated the benefit of targeting areas of late LV activation (the Imaging CRT trial and the TARGET trial).^11^^,^^13^ These show comparable results to our analysis combining the STARTER and the CRT Clinic Trials. The individual results of the 4 trials are summarized in Table 4. Even though there were differences with regard to which of the individual endpoints (echocardiographic, clinical response, or hard endpoints) were significant in the 4 studies, the results are overall congruent and point in the same direction. The rationale for reaching a better clinical outcome by targeting the latest mechanically activated segment is that the most effective resynchronization would be achieved by recruiting the latest contracting free wall segment first, and then spreading activation from this point simultaneously with the septal activation wavefront emerging from the RV electrode. Optimal resynchronization would in turn result in more pronounced reverse remodeling of the left ventricle, which over time will transform into better clinical outcome. It is therefore likely that some of the beneficial effects of optimized resynchronization have a gradual effect, which increases over time during the first years post implant. The magnitude of the effect also depends partly on the prognosis of the control arm who receives standard of care, where concordant LV lead placement may occur fortuitously. Furthermore, starting from the 2013 CRT guidelines, targeting a posterolateral nonapical location (also the most common optimal location in the present meta-analysis) was specifically recommended, and this recommendation may have helped to eliminate “remote” LV lead locations in patients where no imaging is performed.^15^

**Table 4:** Summary of the main results from the 4 major randomized trials for targeting the latest mechanically activated segment

Trial	LV lead placement (optimal/adjacent/distant), %		Reverse remodeling (LVESV reduction ≥15%)		Clinical responder (NYHA class improvement ≥1)†		Freedom from death or heart failure hospitalization after 2 years	
	Intervention	Control	Intervention	Control	Intervention	Control	Intervention	Control
STARTER^12^	30/55/15	12/44/33	57%	35%‡	82%	80%	77%	57%‡
TARGET^11^	61/25/10	45/28/24	70%	55%‡	83%	65%‡	86%	78%‡
Imaging CRT^13^	49/50/1	43/54/2	N/r	N/r	60%	51%‡	78%	80%
CRT Clinic^14^	21/62/17	15/58/27	56%	55%	74%	67%	80%	96%

CRT = cardiac resynchronization therapy; LV = left ventricular; LVESV = left ventricular end-systolic volume; N/r = not reported; NYHA = New York Heart Association classification of heart failure.

† Imaging CRT used a definition that included either NYHA class improvement or ≥10% improvement at 6-min walk test, in absence of heart failure hospitalization or death.

‡ *P* < .05.

Adding these medium-term results for the targeted LV lead strategy is an important step toward a more general recommendation on individualized LV lead placement for CRT therapy. Even though short-term results from the 4 published RCTs generally were positive, the effect on surrogate endpoints such as reverse remodeling were ambiguous, with Imaging CRT and CRT Clinic showing no difference on reverse remodeling between groups. It is, however, well known that agreement between reverse remodeling after CRT and clinical outcome is relatively poor,^16^ and the positive results of the present medium-term follow-up including the CRT Clinic database strengthens the notion that targeting the latest mechanical activation is beneficial. The result was mainly driven by heart failure hospitalizations, but for the subgroup of diseased patients where cause of death was known, there seemed to be strong signal for lower risk of death from heart failure in the intervention group. Considering the substantial number of missing data on cause of death, this finding should be interpreted with caution and will need to be further explored and validated in other studies.

Even though all 4 studies showed a higher proportion of concordant LV leads in the intervention group, the percentage of patients with remote LV leads differed between studies, as did the magnitude of difference between intervention and control groups. This may have affected the results, since the largest differences in actual LV lead position between groups were seen in the TARGET and STARTER studies, which coincides with a positive effect on clinical endpoints within 2 years.

### Identifying the latest mechanically activated segment

Several other strategies for targeted LV lead placement have been suggested, including targeting the latest electrically delayed segment or any of the posterolateral segments without scar.^6^^,^^17^, ^18^, ^19^ Even though all these strategies seem theoretically appealing, it is still unclear if they are equally good, or which is the optimal strategy. Electrical and mechanical activation patterns may vary depending on the underlying substrate for the widened QRS, such as presence and location of myocardial scar, size of the LV cavity, and surface ECG morphology.^20^ In addition, the activation pattern during simultaneous RV pacing may change significantly compared to intrinsic LBBB pattern, thus conferring an additional confounder for targeting the optimal pacing segment.^21^^,^^22^ Only 1 study has so far prospectively compared electrically guided LV lead implantation with mechanically guided LV lead implantation and found no significant difference on clinical endpoints.^23^

### Clinical implications and patient selection regarding ECG criteria

In the absence of other prospectively validated strategies, targeting the latest mechanically activated segment is a reasonable strategy to improve prognosis for CRT recipients for the time being. There are several imaging strategies available to evaluate mechanical activation, including CMR with several subtypes, cardiac computed tomography, and echocardiography using either tissue Doppler or speckle-tracking technique. All methods have their strengths and weaknesses, including feasibility, validation, reproducibility, and temporal resolution.^24^ Differences in mechanical activation times are relatively short, and a high time resolution is therefore essential. Echocardiography-based strain has the best temporal resolution (60–90 fps) and is the only modality that so far has been prospectively validated for CRT regarding segmental strain. CMR-based strain, however, allows for better reproducibility and global strain by CMR has been shown to associate with reverse remodeling.^25^

Observational and randomized trial data have indicated that patients with non-LBBB morphology obtain less benefit from CRT, compared to those with LBBB or strict LBBB fulfilling Strauss’ criteria.^26^^,^^27^ Since LV activation sequence is more unpredictable in non-LBBB, there may be a higher potential gain for individualized LV lead placement in this patient group. We therefore performed sub-analyses to determine if there was a difference in latest activated segment, or a prognostic difference, depending on native ECG morphology. Our results in this respect indicate that there is no such difference and that the distribution of latest activated segments was similar for LBBB and non-LBBB, as was the success rate of placing the LV lead at the intended site in the intervention group.

### Limitations

The present study includes data from 2 separate randomized controlled trials. Even though every effort was made to ensure consistency in each presented variable, the studies did not have a joint case report form or protocol for the original data collection. Therefore, there may be minor discrepancies between the datasets, which have not been detected or corrected for. The total number of patients is limited (n = 289), although this is the largest published analysis so far. Data collection was done during the period 2005–2016. Since then, contemporary guidelines for CRT have changed (eg, QRS duration must now be ≥130 ms instead of ≥120 ms), and optimal medical therapy has evolved to include more classes of drugs. Furthermore, there was a relatively high incidence of ischemic cardiomyopathy in the cohort (53% overall), which may have adversely impacted CRT response in the entire group. These factors could limit the generalizability of our results in a contemporary cohort of CRT-eligible patients. We performed predetermined subgroup analyses stratified for ECG morphology; however, the total number of non-LBBB patients in this cohort was relatively low, and therefore these results should be interpreted with caution. The technical tools nowadays provide better opportunities for targeted lead placement, and if the study were repeated today this could have impacted the results.

## Conclusion

In this patient-level meta-analysis, we show that targeting the LV site of latest mechanical activation in CRT results in improved medium-term survival free from heart failure hospitalizations. This benefit is mainly driven by a reduced risk of hospitalization for heart failure. In the absence of other prospectively validated targeting modalities with proven clinical benefit, it may be reasonable to target the site of latest LV mechanical activation during CRT device implantation.
